# Ulinastatin mitigates protamine-induced hypotension during cardiac surgery: A retrospective observational study using propensity score matching

**DOI:** 10.1097/MD.0000000000045545

**Published:** 2025-11-21

**Authors:** Keisuke Omiya, Yosuke Nakadate, Takeshi Oguchi, Takashi Matsukawa

**Affiliations:** a Department of Anesthesiology, University of Yamanashi, Chuo, Yamanashi, Japan; b Department of Anesthesiology, University of Tsukuba, Tsukuba, Ibaraki, Japan.

**Keywords:** cardiac surgery, heparin, protamine, systemic vascular resistance, ulinastatin

## Abstract

Although protamine induces cardiotoxicity, ulinastatin has been shown to attenuate protamine-induced cardiotoxicity in rats. However, whether ulinastatin alleviates protamine-induced cardiac depression during cardiac surgery remains unclear. This study aimed to evaluate the degree of protamine-induced hypotension in patients undergoing cardiac surgery who received ulinastatin.

This post hoc exploratory analysis included patients undergoing scheduled cardiac surgery. Patients were divided into 2 groups: ulinastatin and control. Patients in the ulinastatin group received 300,000 U of ulinastatin before cardiopulmonary bypass, while those in the control group did not receive ulinastatin. Propensity score matching was performed. Arterial blood pressure (ABP), pulmonary artery pressure, continuous cardiac index, systemic vascular resistance index, and vasopressor use were recorded. The primary outcome was the change in mean ABP after protamine administration.

After propensity score matching, 30 patients were included in each of the control and ulinastatin groups. Patient characteristics were comparable between the 2 groups. The protamine-induced decrease in mean ABP was significantly lower in the ulinastatin group than in the control group (4 [0–9] vs 8 [4–13] mm Hg, median [interquartile range], *P* = .015). There were no significant differences in pulmonary artery pressure and vasopressor use between the groups. While the continuous cardiac index was comparable between the groups after protamine administration, systemic vascular resistance index was significantly higher in the ulinastatin group than in the control group (2090.1 [342.5] vs 1616.2 [644.4] dynes*sec/cm^5^/m^2^, mean [standard deviation], *P* = .022).

Ulinastatin mitigates protamine-induced hypotension, maintaining systemic vascular resistance.

## 1. Introduction

Protamine, a protein isolated from salmon sperm, is used to reverse the anticoagulating effects of heparin during cardiac surgery involving cardiopulmonary bypass (CPB), but it can cause hemodynamic depression.^[1]^ This protamine-induced circulatory suppression is related to 3 mechanisms: myocardial depression caused by tumor necrosis factor-alpha (TNF-α),^[2]^ vasodilation induced by nitric oxide,^[3]^ and pulmonary hypertension mediated by arachidonic acid cascade.^[4]^

Ulinastatin, a human urinary protease inhibitor used clinically to treat circulatory failure, has been shown to reduce protamine-induced cardiotoxicity in studies using rat heart models.^[5]^

However, the effect of ulinastatin on the protamine-induced hypotension remains unclear in the clinical setting.

In this study, we retrospectively investigated the degree of protamine-induced hypotension in patients who underwent cardiac surgery and received ulinastatin treatment.

## 2. Methods

This single-institution retrospective study was approved by the Ethics Committee of the University of Yamanashi (Yamanashi, Japan, protocol number 2799) on March 29, 2024. The Ethics Committee waived the requirement for informed consent because of the retrospective nature of the study. Written informed consent from each individual was omitted by disclosing the information on the website of the Ethics Committee in accordance with the Japanese guidelines. This manuscript adheres to the Strengthening the Reporting of Observational Studies in Epidemiology guidelines.^[6]^

### 2.1. Patient selection

This post hoc exploratory analysis included patients who underwent scheduled cardiac surgery (excluding aortic surgery) using CPB at the University of Yamanashi Hospital (Yamanashi, Japan) between January 16, 2015, and December 27, 2017.

Patients were excluded if they were aged under 18 years, on dialysis, intubated or receiving catecholamine before entering the operating room; underwent rethoracotomy; received ulinastatin at a dose under 300,000 U; or opted out.

Patients were divided into 2 groups according to ulinastatin administration: the ulinastatin and control groups. Patients in the ulinastatin group received 300,000 U of ulinastatin (Mochida Pharmaceutical, Tokyo, Japan) before CPB, while those in the control group did not.

### 2.2. Anesthetic care

The basic anesthesia method was standardized and the cardiac procedures were performed by the same team. In addition to standard monitoring, a radial artery catheter, pulmonary artery catheter, central venous catheter, and transesophageal echocardiography probe were inserted. All patients were intubated and maintained on sevoflurane, midazolam, propofol, remifentanil, fentanyl, and rocuronium. Most patients received methylprednisolone (125–500 mg) before CPB at the discretion of the attending anesthesiologist. The inhaled oxygen concentration was adjusted between 30% and 100% based on blood oxygen saturation. Ringer’s bicarbonate solution and Ringer’s acetate solution with 1% glucose were used as intravenous fluids. Before CPB, heparin of approximately 300 U/kg was administered intravenously, with additional doses given if necessary to maintain an activated clotting time > 480 seconds, measured using a HEMOCHRON Jr (Accriva Diagnostics, Bedford). Mean arterial blood pressure (ABP) was maintained between 50 and 80 mm Hg during CPB. Protamine was administered at approximately 3 mg/kg in 10 minutes to reverse the anticoagulant effect of heparin.

### 2.3. Perioperative variables

Patient characteristics, including age, sex, body mass index, glycated hemoglobin A1c, hematocrit, creatinine, left ventricle ejection fraction, use of methylprednisolone, surgical type, and comorbidities, were collected. ABP, central venous pressure, pulmonary artery pressure (PAP), continuous cardiac index (CCI), and systemic vascular resistance index (SVRI) were also measured before and after protamine administration. The CCI and SVRI were calculated using the Vigilance System (Edwards Life Sciences, Irvine). Furthermore, vasopressors dosage was assessed.

### 2.4. Outcomes

The primary outcome was the protamine-induced decrease in mean ABP. Secondary outcomes were vasopressor use during protamine administration and other hemodynamic parameters.

### 2.5. Statistical analysis

Continuous variables are expressed as mean (standard deviation, SD), or median [interquartile range], and categorical variables are presented as numbers (percentages). Normality was evaluated using the Shapiro–Wilk test. Continuous data were analyzed using the unpaired *t*-test or Mann–Whitney U test, while categorical data were analyzed using the Fisher exact test. Two-sided *P*-values < .05 were considered statistically significant.

Propensity score matching was performed to match the study groups using logistic regression analysis, with the following potential confounders as independent variables: age, sex, body mass index, hypertension, dyslipidaemia, diabetes mellitus, hemoglobin A1c, hematocrit, creatinine, left ventricle ejection fraction, methylprednisolone use, and surgical type. The nearest-neighbor matching method (1:1 ratio) was applied using a caliper width of 0.2 on the logit-transformed propensity score.

The sample size was calculated as follows. To achieve 80% power to detect a 50% difference in the protamine-induced decrease in mean ABP, assuming a SD of 5 mm Hg and an alpha error of 5%, a minimum of 26 patients was required per group.

Missing data were excluded from the analysis. All statistical analyses were performed using GraphPad Prism version 8 for Windows (GraphPad Software, San Diego) and SPSS version 27 (IBM Corp., Armonk).

## 3. Results

**Figure 1.**
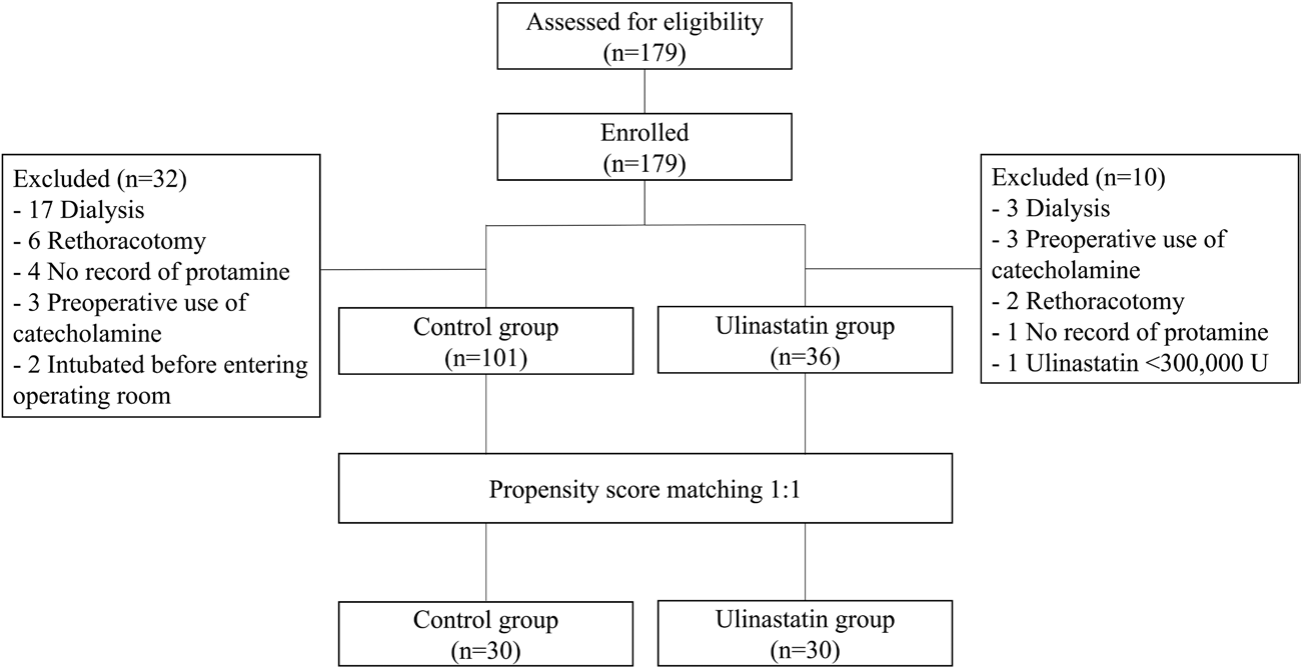
Flow diagram of patient selection.

The patient flow diagram is presented in Figure 1. We enrolled 179 patients in this study. After propensity score matching, 30 patients in each of the control and ulinastatin groups were included in the analysis.

Baseline characteristics were comparable between the 2 groups (Table 1). Hemodynamic outcomes are shown in Table 2. The mean ABP after protamine administration was significantly lower in the control group than in the ulinastatin group (60.4 [8.0] vs 66.7 [9.3] mm Hg, mean [SD], *P* = .002). The decrease in mean ABP from before to after protamine administration was significantly smaller in the ulinastatin group than in the control group (4 [0–9] vs 8 [4–13] mm Hg, median [interquartile range], *P* = .015). There was no significant difference in PAP. While the CCI after protamine administration was comparable between the 2 groups, SVRI was significantly higher in the ulinastatin group than in the control group (2090.1 [342.5] vs 1616.2 [644.4] dynes*sec/cm^5^/m^2^, *P* = .022). Some data on the CCI and SVRI were missing. Therefore, the number of patients with available CCI and SVRI data was 13 in the control group and 14 in the ulinastatin group (Supplemental Digital Content, https://links.lww.com/MD/Q737). There was no significant difference in vasopressor use (Table 3).

**Table 1:** Patient characteristics before and after propensity score matching.

	Total cohort			Propensity score-matched cohort		
	Control (n = 101)	Ulinastatin (n = 36)	*P* value	Control (n = 30)	Ulinastatin (n = 30)	*P* value
Age (yr)	73 [66–79]	73 [63–81]	.73	73 [66–75]	73 [62–82]	.50
Female-n (%)	30 (30)	15 (42)	.22	11 (37)	10 (33)	>.99
BMI (kg/m^2^)	23.1 (3.1)	23.3 (3.7)	.70	23.0 (3.1)	23.1 (3.9)	.94
Hypertension-n (%)	76 (75)	30 (83)	.36	23 (77)	24 (80)	>.99
Dyslipidaemia-n (%)	52 (51)	17 (47)	.70	13 (43)	14 (47)	>.99
DM-n (%)	31 (31)	8 (22)	.39	6 (20)	7 (23)	>.99
HbA1c (%)	5.8 [5.5–6.5]	5.7 [5.4–6.2]	.28	5.7 [5.4–6.2]	5.7 [5.4–6.2]	.64
Hct (%)	38.5 [34.4–41.9]	36.8 [33.2–41.7]	.35	38.8 [36.0–36.3]	36.5 [33.8–41.8]	.25
Creatinine (mg/dL)	0.92 [0.78–1.14]	0.92 [0.76–1.14]	.97	0.92 [0.77–1.10]	0.95 [0.78–1.15]	.50
LVEF (%)	65.0 [54.0–72.5]	66.0 [54.0–75.0]	.68	67.5 [56.0–74.0]	64.5 [54.5–75.0]	.59
Methylprednisolone before CPB-n (%)	69 (68)	35 (97)	.0002	30 (100)	29 (97)	>.99
Surgical type						
Valve surgery (1 valve)-n (%)	50 (49)	16 (45)	.79	19 (63)	15 (50)	.43
Valve surgery (>1 valve)-n (%)	21 (21)	9 (25)	.64	5 (17)	8 (27)	.53
CABG-n (%)	22 (22)	8 (22)	>.99	5 (17)	5 (17)	>.99
CABG + valve surgery-n (%)	7 (7)	3 (8)	.72	1 (3)	2 (6)	>.99
Others (%)	1 (1)	0 (0)	>.99	0 (0)	0 (0)	-

Data are expressed as mean (standard deviation), median [interquartile range], or number of patients (%).

BMI = body mass index, CABG = coronary artery bypass graft, CPB = cardiopulmonary bypass, DM = diabetes mellitus, HbA1c = glycated hemoglobin A1c, Hct = hematocrit, LVEF = left ventricle ejection fraction.

**Table 2:** Hemodynamic outcomes.

	Propensity score-matched cohort		
	Control (n = 30)	Ulinastatin (n = 30)	*P* value
Data at the start of surgery			
Mean ABP (mm Hg)	67.5 (9.9)	71.9 (9.3)	.08
HR (bpm)	69 [61–85]	64 [57–77]	.24
Mean PAP (mm Hg)	21.4 (5.3)	23.4 (5.0)	.14
CVP (mm Hg)	9 [7–11]	9 [7–12]	.83
CCI (L/min/m^2^)	2.5 [2.2–3.1]	2.6 [2.2–3.0]	.90
SVRI (dynes × sec/cm^5^/m^2^)	1799.9 (547.9)	1903.6 (379.6)	.50
Data before protamine administration			
Mean ABP (mm Hg)	70 [67–74]	71 [66–78]	.53
HR (bpm)	83.1 (8.5)	82.7 (9.7)	.84
Mean PAP (mm Hg)	23.2 (5.4)	23.0 (3.9)	.89
CVP (mm Hg)	8.6 (2.9)	8.2 (3.1)	.63
CCI (L/min/m^2^)	2.1 [1.8–3.0]	2.0 [1.7–2.3]	.32
SVRI (dynes × sec/cm^5^/m^2^)	2309.8 (874.4)	2464.2 (435.1)	.56
Data after protamine administration			
Mean ABP (mm Hg)	60.4 (8.0)	67.7 (9.3)	.002
HR (bpm)	80 [79–86]	80 [80–89]	.68
Mean PAP (mm Hg)	21.4 (5.6)	21.4 (4.3)	.98
CVP (mm Hg)	8.4 (2.7)	7.7 (2.7)	.30
CCI (L/min/m^2^)	2.4 [2.2–3.6]	2.4 [2.1–2.6]	.43
SVRI (dynes × sec/cm^5^/m^2^)	1616.2 (644.4)	2090.1 (342.5)	.022
Change before and after protamine administration			
Decrease in mean ABP (mm Hg)	8 [4–13]	4 [0–9]	.015
Decrease in mean PAP (mm Hg)	2 [0–4]	1 [0–3]	.42
Increase in CCI (L/min/m^2^)	0.2 [0.1–0.5]	0.3 [0.1–0.4]	.86
Decrease in SVRI (dynes × sec/cm^5^/m^2^)	693.6 (479.4)	374.1 (370.0)	.059

Data are expressed as mean (standard deviation) or median [interquartile range].

ABP = arterial blood pressure, CCI = continuous cardiac index, CVP = central venous pressure, HR = heart rate, PAP = pulmonary artery pressure, SVRI = systemic vascular resistance index.

**Table 3:** Use of vasopressors and dilators during protamine administration.

	Propensity score-matched cohort		
	Control (n = 30)	Ulinastatin (n = 30)	*P* value
Average of dopamine (µg/kg/min)	3.2 [2.2–4.7]	2.9 [2.1–3.8]	.30
Average of dobutamine (µg/kg/min)	0.0 [0.0–2.3]	0.0 [0.0–0.7]	.16
Average of norepinephrine (µg/kg/min)	0.0 [0.0–0.0]	0.0 [0.0–0.0]	.91
Phenylephrine (mg)	0.0 [0.0–0.0]	0.0 [0.0–0.0]	.17
Average of nitroglycerin (µg/kg/min)	0.41 [0.26–0.50]	0.50 [0.28–0.71]	.09
Average of nicardipine (µg/kg/min)	0.0 [0.0–0.0]	0.0 [0.0–0.0]	>.99

Data are expressed as median [interquartile range].

The original source datasets are available in Supplemental Digital Content, https://links.lww.com/MD/Q737.

## 4. Discussion

The main findings of our study are that ulinastatin mitigated the protamine-induced decrease in ABP and maintained SVRI. However, protamine administration did not affect the CCI and PAP.

Although we hypothesized that ulinastatin mitigates protamine-induced depression of cardiac contractility, it may prevent protamine-induced vasodilation. Protamine itself in particular might affect systemic vascular resistance, not pulmonary vascular resistance, after weaning from CPB.^[7]^ Ulinastatin has been shown to suppress the production of TNF-α^[8,9]^ and to reduce protamine-induced cardiotoxicity in isolated rat hearts via TNF-α inhibition.^[5]^ However, ulinastatin did not increase the CCI in the present study. This discrepancy may be attributed to differences in the route and timing of administration. In the present study, ulinastatin was administered intravenously before CPB, whereas in the rat heart study, it was administered directly via the perfusate.

Protamine is a polypeptide rich in arginine, a precursor of nitric oxide.^[3]^ In an isolated rabbit vascular model, protamine induced vasodilation by extending the vascular endothelium via nitric oxide.^[3]^ In an in vivo dog experiment, the protamine-induced decrease in BP was mitigated by a nitric oxide inhibitor.^[10]^ In mouse microglial cells, ulinastatin inhibited nitric oxide production by suppressing inducible nitric oxide synthase expression.^[11]^ In the present study, ulinastatin may have inhibited the vasodilatory effect of protamine by suppressing nitric oxide production, thereby mitigating the decrease in ABP.

Additionally, ulinastatin has been reported to exert pulmonary protective and anti-inflammatory effects in patients undergoing cardiac surgery.^[12,13]^ Furthermore, in patients undergoing abdominal aortic aneurysmectomy, ulinastatin has been shown to prevent an increase in PAP after aortic unclamping.^[14]^ However, ulinastatin did not decrease PAP in the present study. Protamine has been shown to induce pulmonary hypertension through the arachidonic acid cascade.^[4]^ However, we did not investigate substances associated with the arachidonic acid cascade. Further studies are required to clarify the mechanism of action of ulinastatin treatment.

This study has several limitations. First, the sample size was small. A total of 36 patients per group would have been required, assuming a SD of 450 dynes × sec/cm^5^/m^2^, a power of 0.8, and an alpha error of 5%, to detect a significance 300 dynes × sec/cm^5^/m^2^ difference in the protamine-induced decrease in SVRI between the control and ulinastatin groups. Second, steroids might mitigate protamine-induced hypotension because glucocorticoids reduce TNF-α, arachidonic acid, and nitric oxide synthase,^[7,15–17]^ which could influence the impact of ulinastatin treatment on cardiac decompression. Although most patients in the present study received methylprednisolone before CPB after propensity score matching, the routine use of methylprednisolone in patients undergoing CPB has not been recommended recently.^[18]^ Therefore, further clinical studies excluding steroids use are required.

## 5. Conclusion

Intravenous administration of ulinastatin before CPB mitigated the protamine-induced hypotension and maintained systemic vascular resistance.

## Acknowledgments

We would like to thank Editage (http://www.editage.com) for English language editing.

## Author contributions

**Conceptualization:** Keisuke Omiya, Takashi Matsukawa.

**Data curation:** Keisuke Omiya, Yosuke Nakadate.

**Formal analysis:** Keisuke Omiya, Yosuke Nakadate, Takeshi Oguchi.

**Investigation:** Keisuke Omiya, Yosuke Nakadate.

**Methodology:** Keisuke Omiya, Yosuke Nakadate.

**Supervision:** Takashi Matsukawa.

**Validation:** Keisuke Omiya, Yosuke Nakadate, Takeshi Oguchi.

**Visualization:** Keisuke Omiya.

**Writing – original draft:** Keisuke Omiya, Yosuke Nakadate.

**Writing – review & editing:** Takeshi Oguchi, Takashi Matsukawa.

## Supplementary Material


